# Role of fetal pulmonary artery Doppler parameters for predicting neonatal respiratory distress syndrome: A systematic review and meta-analysis of diagnostic accuracy

**DOI:** 10.1016/j.eurox.2026.100494

**Published:** 2026-09-16

**Authors:** Potsanop Kassayanan, Kasidis Nontaprom, Atiwit Threeratananont, Nathaporn Thitinan, Monchai Suntipap

**Affiliations:** a Faculty of Medicine, Srinakharinwirot University, Ongkharak, Nakhon Nayok, 26120, Thailand; b Department of Obstetrics and Gynecology, Faculty of Medicine, Srinakharinwirot University, Ongkharak, Nakhon Nayok, 26120, Thailand

**Keywords:** Acceleration time-to-ejection time ratio, Diagnostic accuracy, Fetal pulmonary artery Doppler, Meta-analysis, Neonatal respiratory distress syndrome, Pulsatility index, Systematic review

## Abstract

**Objective:**

To evaluate the diagnostic accuracy of fetal pulmonary artery Doppler parameters for predicting neonatal respiratory distress syndrome (NRDS).

**Data sources:**

MEDLINE, Scopus, Embase, and Google Scholar were searched through March 30, 2026.

**Study eligibility criteria:**

Studies reporting a fetal pulmonary artery Doppler parameter and diagnostic accuracy or continuous data for predicting NRDS were eligible.

**Methods:**

This systematic review followed PRISMA-DTA guidelines, and risk of bias was assessed with QUADAS-2. Pooled sensitivity, specificity, and likelihood ratios were estimated using a bivariate random-effects model. Hierarchical summary receiver operating characteristic curves estimate the area under the curve (AUC). Subgroup, sensitivity, and meta-regression analyses explored heterogeneity.

**Results:**

Twenty-eight studies (n = 3576) were included. Acceleration time-to-ejection time ratio (AT/ET; 21 studies) demonstrated the most robust performance (sensitivity 90%, specificity 87%, AUC 0.94). Peak systolic velocity (PSV) showed a numerically high but highly imprecise performance (sensitivity 85%, specificity 93%, AUC 0.94), followed by pulsatility index (PI; AUC 0.89) and resistance index (RI; AUC 0.86). PI and RI were significantly higher, and AT/ET, PSV, and mean velocity significantly lower, in fetuses whose neonates developed NRDS. Evidence for systolic-to-diastolic ratio, end-diastolic velocity, and mean velocity remained insufficient for pooled diagnostic accuracy analysis. Substantial heterogeneity was observed in the pooled diagnostic accuracy analyses.

**Conclusions:**

Fetal pulmonary artery Doppler ultrasonography, particularly AT/ET, PI, and RI, shows promising diagnostic performance for predicting NRDS, with AT/ET most consistent across gestational age strata. Although PSV yielded a numerically high AUC, its limited study number and imprecision preclude firm conclusions regarding diagnostic value.

## 1 Introduction

Neonatal respiratory distress syndrome (NRDS) is one of the most common respiratory complications arising from incomplete development of type II pneumocytes, resulting in pulmonary surfactant insufficiency [1]. The prevalence of NRDS varies according to gestational age (GA), reaching up to 45% in early preterm births (PTB) and decreasing with advancing GA [2,3]. NRDS accounts for 11.9–45.1% of neonatal intensive care unit (NICU) admissions [[4], [5], [6]].

Current management of NRDS is based on antenatal corticosteroid administration to promote fetal lung maturation, as well as postnatal respiratory support and exogenous surfactant therapy. However, prenatal risk stratification remains challenging, as current assessment still relies largely on GA. Although the lecithin-to-sphingomyelin ratio has shown good diagnostic performance, its use in clinical practice remains limited because of its invasiveness [7]. In addition, ultrasonographic parameters, such as fetal lung volumetry and texture analysis, have not been established as standard tools for predicting NRDS [8,9].

Fetal pulmonary artery Doppler ultrasonography has been proposed as a potential tool for predicting NRDS because it may reflect fetal pulmonary vascular development and lung maturation before delivery [10]. A wide range of Doppler parameters have been explored, including the pulsatility index (PI), resistance index (RI), acceleration time-to-ejection time ratio (AT/ET), systolic-to-diastolic ratio (S/D), peak systolic velocity (PSV), end-diastolic velocity (EDV), mean velocity (MV), and mean pulmonary artery pressure (PAP). However, a comprehensive analysis of the diagnostic performance of each parameter has not yet been performed.

Therefore, this systematic review and meta-analysis (SRMA) aimed to evaluate the diagnostic performance of fetal pulmonary artery Doppler parameters and summarize the evidence for predicting NRDS.

## 2 Methods

### 2.1 Study design and registration

This study was conducted in accordance with the Preferred Reporting Items for Systematic Reviews and Meta-Analyses for Diagnostic Test Accuracy (PRISMA-DTA) guidelines [11]. The study was prospectively registered in the PROSPERO database (registration number: CRD420251150745; registered prior to data extraction).

### 2.2 Information sources and search strategy

Three electronic bibliographic databases, MEDLINE, Scopus, and Embase, were systematically searched, along with manual searching via Google Scholar, from inception to March 30, 2026. The search strategy was developed based on the PIRD framework [12] using relevant search terms related to pregnant women, fetus, pulmonary artery Doppler indices, and neonatal respiratory distress syndrome (Supplementary Table S1.1-S1.3). Subsequently, titles and abstracts were screened independently by two reviewers (P.K. and K.N.).

### 2.3 Study selection

All eligible studies were independently assessed for inclusion by two reviewers (P.K. and K.N.) through full-text review. The inclusion criteria were studies that assessed at least one fetal pulmonary artery Doppler index and reported outcomes of interest, including diagnostic performance or continuous outcomes. Studies published in non-English languages that could not be translated using Google Translate or artificial intelligence-assisted tools such as Claude AI, as well as studies with insufficient data for pooling the outcomes of interest despite three attempts to contact the authors at two-week intervals, were excluded. Any disagreements were discussed and resolved through consultation with a third reviewer (M.S.).

### 2.4 Data extraction

Two reviewers independently performed data extraction using a Microsoft Excel form. Extracted data included study characteristics, participant demographics, neonatal outcomes, diagnostic accuracy data (cutoff values, true/false positives and negatives, sensitivity, specificity, LR+, and LR−), and continuous outcomes (mean and standard deviation in NRDS and non-NRDS groups). Data reported as median with range or interquartile range were converted to mean and standard deviation using the method described by Wan et al. [13]. When complete 2 × 2 contingency data were not directly reported, cell counts were reconstructed from the reported diagnostic accuracy measures and available group sizes for inclusion in the diagnostic meta-analysis.

### 2.5 Risk of bias and publication bias assessment

The quality assessment was independently performed by two reviewers (P.K. and K.N.) using the Quality Assessment of Diagnostic Accuracy Studies-2 (QUADAS-2) tool [14], which assesses risk of bias across four domains and applicability concerns across the first three domains. Each section was judged as having a high, unclear, or low risk of bias. Publication bias was assessed using Deeks’ funnel plot, and any observed asymmetry was further explored to determine whether it was due to heterogeneity or publication bias. Any disagreements were discussed and resolved through consultation with a third reviewer (M.S.).

### 2.6 Statistical analysis

Diagnostic accuracy outcomes were synthesized using a bivariate random-effects model to jointly estimate pooled sensitivity and specificity, together with LR+, LR−, and diagnostic odds ratios. Hierarchical summary receiver operating characteristic curves were constructed to estimate the area under the curve. For continuous outcomes, pooled mean differences with 95% confidence intervals were estimated using a random-effects model. Mean differences were calculated as values in the non-RDS group minus those in the RDS group. Heterogeneity was assessed using the I² statistic, with I² greater than 50% indicating substantial heterogeneity. Potential sources of heterogeneity were explored using meta-regression with fetal population category, geographic region, sample size, NRDS prevalence, and antenatal corticosteroid use as study-level covariates. Meta-regression analyses were considered exploratory because the number of studies contributing to each parameter-specific model was limited, which may have reduced statistical power to detect associations with diagnostic performance. Subgroup analyses were performed for parameters with sufficient contributing studies according to fetal population category and geographic region, where data permitted. Sensitivity analyses were performed by sequential exclusion of studies with extreme sensitivity estimates and by exclusion of identified outlier studies in the continuous outcome analyses. All analyses were performed using Stata version 18.0 (StataCorp, College Station, TX, USA). A p-value < 0.05 was considered statistically significant, except for Deeks’ test for publication bias, for which a p-value < 0.10 was considered significant.

## 3 Results

### 3.1 Study selection

**Fig. 1:**
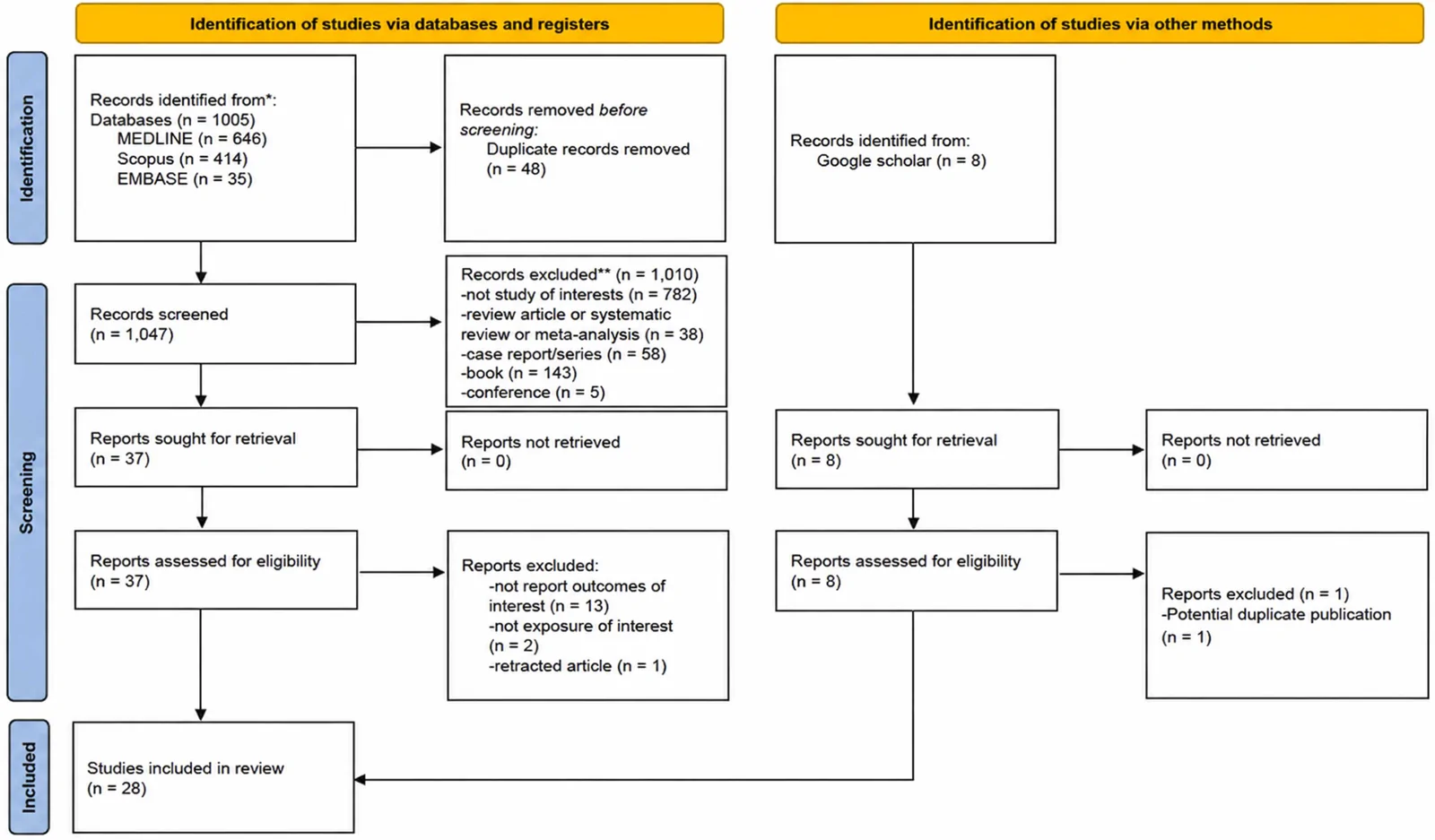
PRISMA flow diagram of study selection.

A total of 1095 studies were identified. After removing 48 duplicate studies, 1047 studies remained for title and abstract screening. Of these, 1010 studies were excluded. Then, 37 studies were assessed for eligibility. Sixteen studies were excluded for the following reasons: 13 did not report the outcomes of interest, 2 did not meet the exposure criteria, and 1 was a retracted study. In addition, 8 studies were identified through manual searching via Google Scholar, but one study was excluded due to potential duplicate publication. Finally, 28 studies met the inclusion criteria and were included in this analysis. The PRISMA flow diagram is shown in Fig. 1, and the details of excluded studies are provided in Supplementary Table S2.

### 3.2 Study characteristics

A total of 28 studies published between 2013 and 2026 were included, comprising 3576 pregnancies, including 818 neonates diagnosed with NRDS [8,[15], [16], [17], [18], [19], [20], [21], [22], [23], [24], [25], [26], [27], [28], [29], [30], [31], [32], [33], [34], [35], [36], [37], [38], [39], [40], [41]]. The included studies originated predominantly from Egypt and India. Most studies employed a prospective cross-sectional or prospective cohort design. Sample sizes ranged from 25 to 698 participants. GA at the time of Doppler assessment ranged from 24 to 40 weeks. Across all included studies, PI, RI, AT/ET, S/D, PSV, EDV, and MV were evaluated in 20, 26, 23, 7, 14, 4, and 3 studies, respectively. No included study reported PAP for either diagnostic or continuous outcomes. For diagnostic accuracy analysis, 10 PI studies, 15 RI studies, 21 AT/ET studies, 2 S/D studies, 5 PSV studies, and 2 MV studies contributed relevant data, whereas no diagnostic accuracy data were available for EDV. For continuous outcome analysis, the numbers were 20 for PI, 26 for RI, 23 for AT/ET, 7 for S/D, 13 for PSV, 4 for EDV, and 3 for MV. Detailed characteristics of the included studies are presented in Table 1, Table 2, Table 3**, and** study-level Doppler details are presented in Supplementary Tables S3 and S4.

**Table 1:** General characteristics of included studies.

Author, year	Country	Design	Sample size, n	NRDS/ non- NRDS	Fetal population	Ultrasound (type, name, method)	Doppler parameters	Neonatal RDS definition	Reproducibility reported (ICC, inter-/intra-observer)
Kim et al. [15]	South Korea	Prospective cohort	42	11 / 31	Preterm, unspecified	2D, Acuson Sequoia 512, Siemens, Germany	PI, RI, AT/ET, S/D, PSV	RD + FiO2 > 0.4 + typical CXR; other causes excluded	Yes (ICC inter 0.86; intra 0.97)
Guan et al.[16]	China	Prospective cohort	43	14 / 29	Preterm, unspecified	2D, Acuson Sequoia 512, Siemens Medical Solutions, Mountain View, CA	PI, RI, AT/ET, PSV, EDV, MV	RD O2 requirement + surfactant response ± typical CXR	Yes (intraobserver reproducibility only 4.4–16.0% for each parameters)
Laban et al. [8]	Egypt	Cross-sectional	80	11 / 69	Term	2D, Voluson E6 (GE Healthcare, Waukesha, WI, USA)	RI	NR	NR
Moety et al. [17]	Egypt	Prospective cross-sectional	698	55 / 643	Mixed	2D, Voluson 730 (GE Healthcare Austria GmbH, Seoul, South Korea)	PI, RI, AT/ET, S/D, PSV	RD + FiO2 > = 0.4 at least 24 h + typical CXR	NR
Laban et al. [18]	Egypt	Cross-sectional	80	37 / 43	Preterm, unspecified	2D, Voluson E6 (GE Healthcare, Waukesha, WI, USA)	RI	Vermont Oxford criteria	NR
Buke et al. [19]	Turkey	Prospective cohort	105	22 / 83	Preterm, unspecified	2D, Mindray DC-7 (Shenzhen Mindray Bio-medical Electronics Co., Shenzhen, China)	PI, RI, AT/ET, PSV, EDV, MV	RD + FiO2 > 0.4 + typical CXR; other causes excluded	NR
Abdelhamid et al. [20]	Egypt	Prospective cross-sectional	40	9 / 31	Mixed	NR	PI, RI, AT/ET	At least 2/3 of RD + FiO2 > 0.4 for > 24 h; typical CXR; surfactant response	NR
Alsheikh et al. [21]	Egypt	Prospective study	120	16 / 104	Term	NR	PI, RI, AT/ET, PSV	RD only	NR
Kandil et al. [22]	Egypt	Prospective cohort	120	19 / 101	Preterm, unspecified	2D, Ultrasound GE Healthcare	RI	NR	NR
Khalifa et al. [23]	Egypt	Prospective cross-sectional	143	38 / 105	Mixed	2D, Medison SonoAce X6	PI, RI, AT/ET	RD + lung ultrasound	NR
Vafaei et al. [24]	Iran	Prospective cohort	25	8 / 17	Early preterm	2D, GE Voluson E6 (GE Healthcare)	PI, RI, AT/ET	At least 2/3 of RD + O2 requirement for > 24 h; typical CXR; surfactant administration	NR
Elrashedy et al. [25]	Egypt	Prospective cross-sectional	100	13 / 87	Term	NR	PI, RI, AT/ET, S/D, PSV	NR	NR
Guirguis et al. [26]	Egypt	Prospective cross-sectional	60	26 / 34	Mixed	NR	PI, RI, AT/ET	RD + O2 requirement for > 24 h + typical CXR	NR
Hassan et al. [27]	Egypt	Prospective	70	26 / 44	Mixed	2D, Acuson Juniper, Siemens, USA	PI, RI, AT/ET, PSV	RD + FiO2 > = 0.4 at least 24 h + typical CXR	NR
Taha et al. [28]	Egypt	Prospective observational	100	13 / 87	Mixed	2D, Samsung Medison H60, Korea	PI, RI, AT/ET, S/D, PSV	Typical CXR + surfactant administration	NR
Yadav et al. [29]	India	Prospective cross-sectional	75	15 / 60	Late preterm	2D, Philips Epiq 7 G ultrasound	PI, RI, AT/ET, S/D, PSV, EDV	NR	NR
Eldeeb et al. [30]	Egypt	Prospective	147	59 / 88	Preterm, unspecified	2D, Voluson E6 (GE Healthcare, Waukesha, WI, USA)	RI	At least 2/3 of RD + O2 need for > 24 h; typical CXR; surfactant response	NR
Hawas et al. [31]	Egypt	Prospective observational	200	26 / 174	Mixed	2D, Samsung Medison H60, Korea	RI	NR	NR
Bedeer et al. [32]	Egypt	Prospective cohort	100	11 / 89	Term	2D, Voluson S10 ultrasound machine	PI, RI, AT/ET, S/D, PSV	RD + typical CXR + arterial blood gas shows hypoxemia and hypercapnia	NR
Ozturk et al. [33]	Turkey	Case series	25	6 / 19	Preterm, unspecified	2D, Voluson E8 (GE Healthcare, Little Chalfont, UK)	AT/ET	RD + FiO2 > 0.4 + typical CXR; other causes excluded	NR
Seth et al. [34]	India	Prospective observational	62	28 / 34	Preterm, unspecified	NR	PI, RI, AT/ET, PSV, EDV, MV	RD + O2 requirement + typical CXR	NR
Tharwat et al. [35]	Egypt	Cross-sectional	200	113 / 87	Mixed	2D, Voluson S6	PI, RI, AT/ET, PSV	RD + typical CXR	NR
Agrawal et al. [36]	India	Prospective observational	170	39 / 131	Mixed	NR	PI, RI, AT/ET, PSV	At least 2/3 of RD + O2 need for > 24 h; typical CXR; surfactant response	NR
Bhagat et al. [37]	India	Prospective cross-sectional	50	20 / 30	Preterm, unspecified	GE Voluson E8 and GE Logiq P7 ultrasound	PI, RI, AT/ET	RD + O2 requirement for > 24 h + typical CXR	NR
Fasad et al. [38]	Egypt	Prospective study	100	12 / 88	Mixed	Edan U2 ultrasound	RI, AT/ET	NR	NR
Kumar et al. [39]	India	Prospective observational	277	68/ 209	Mixed	NR	PI, RI, AT/ET, S/D, PSV	RD + FiO2 > 0.4 for > 24 h + typical CXR	NR
Saikia et al. [40]	India	Prospective observational	175	82 / 93	Preterm, unspecified	2D, Samsung RS80A Prestige ultrasound machine	AT/ET	NR	NR
Ibrahim et al. [41]	Egypt	Cross-sectional	169	21 / 148	Term	NR	PI, RI, AT/ET	NR	NR

Abbreviations: AT/ET = acceleration time-to-ejection time ratio; CXR = chest X-ray; EDV = end-diastolic velocity; FiO2 = fraction of inspired oxygen; ICC = intraclass correlation coefficient; MV = mean velocity; NR = not reported; NRDS = neonatal respiratory distress syndrome; PI = pulsatility index; PSV = peak systolic velocity; RD = respiratory distress; RI = resistance index; S/D = systolic-to-diastolic ratio.

**Table 2:** Demographic and obstetric characteristics of included studies.

Author, year	Sample size	Population (GA)	Mean age (years)	BMI (kg/m²)	Nulliparous (%)	Mean GA at delivery (weeks)	Steroid n (%)	Cesarean delivery (%)	Preterm birth (%)	Mean birth body weight (g)	APGAR score at 1 min	APGAR score at 5 min	NICU admission (%)
Kim et al. [15]	42	Preterm (24–36 wks)	35.00 (8.48)	NR	48.60	30.86 (8.71)	86.50	64.90	NR	1473.30 (1820.10)	NR	NR	88.10
Guan et al. [16]	43	Preterm (<37 wks)	29.07 (5.87)	NR	53.40	32.85 (2.84)	55.81	79.06	100.00	2166.62 (639.92)	NR	NR	72.09
Laban et al. [8]	80	Term (37–40 wks)	NR	NR	NR	38.67 (0.76)	NR	29.66	0.00	NR	NR	8.67 (0.75)	NR
Moety et al. [17]	698	Mixed (34.0–38^+ 6^ wks)	28.26 (5.25)	NR	29.66	37.30 (1.69)	NR	43.12	NR	3005.77 (1165.41)	6.75 (1.03)	8.84 (1.00)	13.04
Laban et al. [18]	80	Preterm (32–36 wks)	28.56 (4.45)	NR	NR	33.56 (1.11)	NR	43.70	100.00	2645.00 (240.50)	4.56 (0.63)	5.79 (0.43)	63.00
Buke et al. [19]	105	Preterm (<37 wks)	25.61 (5.71)	NR	0.00	34.19 (1.96)	16.19	54.30	100.00	2534.00 (629.00)	NR	NR	32.30
Abdelhamid et al. [20]	40	Mixed (28–40 wks)	26.51 (6.19)	NR	25.00	36.5 (2.78)	NR	NR	NR	2847.76 (629.17)	NR	NR	30.00
Alsheikh et al. [21]	120	Term	26.50 (5.10)	NR	NR	38.4 (0.9)	NR	NR	0.00	3159.00 (364.00)	NR	NR	NR
Kandil et al. [22]	120	Preterm (30–37 wks)	27.98 (4.37)	NR	25.83	35.45 (1.72)	NR	NR	100.00	NR	NR	NR	NR
Khalifa et al. [23]	143	Mixed (32–40 wks)	29.50 (6.20)	26.60 (3.70)	NR	36.5 (1.7)	34.97	54.50	NR	2757.48 (546.97)	NR	6.23 (1.43)	32.20
Vafaei et al. [24]	25	Preterm (28–34 wks)	NR	NR	NR	30.58 (1.1)	NR	NR	100.00	1930.05 (274.75)	NR	NR	NR
Elrashedy et al. [25]	100	Term (38–39 wks)	25.76 (3.19)	NR	NR	NR	NR	100.00	0.00	3340.43 (463.01)	7.48 (1.94)	8.40 (1.13)	NR
Guirguis et al. [26]	60	Mixed (28–40 wks)	NR	NR	NR	36.80 (3.15)	NR	NR	NR	NR	NR	NR	NR
Hassan et al. [27]	70	Mixed (34–38 wks)	31.56 (6.31)	NR	42.90	35.41 (0.97)	NR	58.60	78.60	2595.57 (521.69)	6.93 (2.97)	7.68 (2.42)	41.43
Taha et al. [28]	100	Mixed (34–40 wks)	28.61 (6.00)	NR	21.00	35.7 (1.18)	100.00	100.00	NR	NR	NR	NR	NR
Yadav et al. [29]	75	Late preterm (34–36^+6^ wks)	NR	NR	NR	NR	0.00	NR	NR	NR	7.21	7.88	NR
Eldeeb et al. [30]	147	Preterm (32–36 wks)	29.87 (5.91)	NR	NR	33.79 (1.54)	NR	49.66	100.00	2213.33 (479.08)	6.49 (2.70)	7.00 (2.46)	40.14
Hawas et al. [31]	200	Mixed (36–40 wks)	26.96 (4.41)	NR	NR	37.84 (1.22)	NR	58.00	NR	3395.00 (229.00)	NR	NR	13.00
Bedeer et al. [32]	100	Term (37–38 wks)	22.90 (2.10)	NR	NR	37.16 (0.38)	0.00	65.00	0.00	2831.27 (277.84)	6.55 (0.52)	8.50 (0.54)	40.00
Ozturk et al. [33]	25	Preterm (<37 wks)	32.40 (5.16)	28.79 (4.13)	48.00	NR	NR	NR	100.00	NR	NR	NR	32.00
Seth et al. [34]	62	Preterm (<37 wks)	25.04 (3.53)	NR	NR	33.6 (1.63)	NR	NR	NR	2136.30 (183.01)	NR	NR	NR
Tharwat et al. [35]	200	Mixed (32–39 wks)	28.80 (4.90)	NR	7.00	35.1 (2.1)	NR	74.50	76.00	NR	NR	7.74 (1.38)	56.50
Agrawal et al. [36]	170	Mixed (28–40 wks)	NR	NR	6.40	NR	NR	NR	25.88	NR	NR	NR	NR
Bhagat et al. [37]	50	Preterm (30–36 wks)	26.40 (3.50)	NR	NR	33.82 (1.36)	NR	64.00	100.00	2181.00 (221.96)	NR	NR	52.00
Fasad et al. [38]	100	Mixed (34–40 wks)	25.61 (5.19)	NR	8.00	36.76 (1.27)	NR	NR	NR	NR	NR	NR	NR
Kumar et al. [39]	277	Mixed (>28 wks)	27.40 (4.10)	NR	71.80	NR	NR	NR	33.94	2750.00 (450.00)	7.50 (1.20)	8.50 (1.00)	NR
Saikia et al. [40]	175	Preterm (<37 wks)	NR	NR	NR	NR	0.00	NR	100.00	NR	NR	NR	NR
Ibrahim et al. [41]	169	Term (>36 wks)	27.64 (3.41)	NR	NR	37.41	52.70	54.40	0.00	NR	NR	NR	NR

Abbreviations: GA = gestational age; BMI = body mass index; NR = not reported.

**Table 3:** Summary of study-level diagnostic accuracy data by fetal pulmonary artery Doppler marker for prediction of neonatal respiratory distress syndrome.

Doppler marker	No. of studies	Total participants	Cutoff range	Sensitivity range, %	Specificity range, %
Pulsatility index	10 [17,21,23,[27], [28], [29],^32^,^35^,^37^,^39^]	1833	1.79–2.47	69.1–100.0	34.5–100.0
Resistance index	15 [8,17,18,21,23,[27], [28], [29], [30],^32^,^34^,^35^,[37], [38], [39]]	2302	0.74–0.90	53.85–100.0	43.5–100.0
Acceleration time-to-ejection time ratio	21 [16,17,[19], [20], [21],^23^,[25], [26], [27], [28], [29],[32], [33], [34], [35], [36], [37], [38], [39], [40], [41]]	2882	0.21–0.48	71.4–100.0	20.3–100.0
Systolic-to-diastolic ratio	2 [28,32]	200	6.93–7.00	53.85–59.60	54.4–60.92
Peak systolic velocity	5 [16,21,28,32,34]	425	70.00–78.5	35.7–100.0	43.68–100.0
Mean velocity	2 [16,34]	105	25.50	21.4–70.0	65.0–96.6

Abbreviations: AT/ET, acceleration time-to-ejection time ratio; NRDS, neonatal respiratory distress syndrome.

Note: This table summarizes study-level diagnostic accuracy patterns by Doppler marker. Detailed study-level thresholds, 2 × 2 data, and sensitivity/specificity estimates are provided in Supplementary Table S3. Some studies contributed to more than one Doppler marker category.

### 3.3 Diagnostic performance of fetal pulmonary artery Doppler for PI

**Fig. 2:**
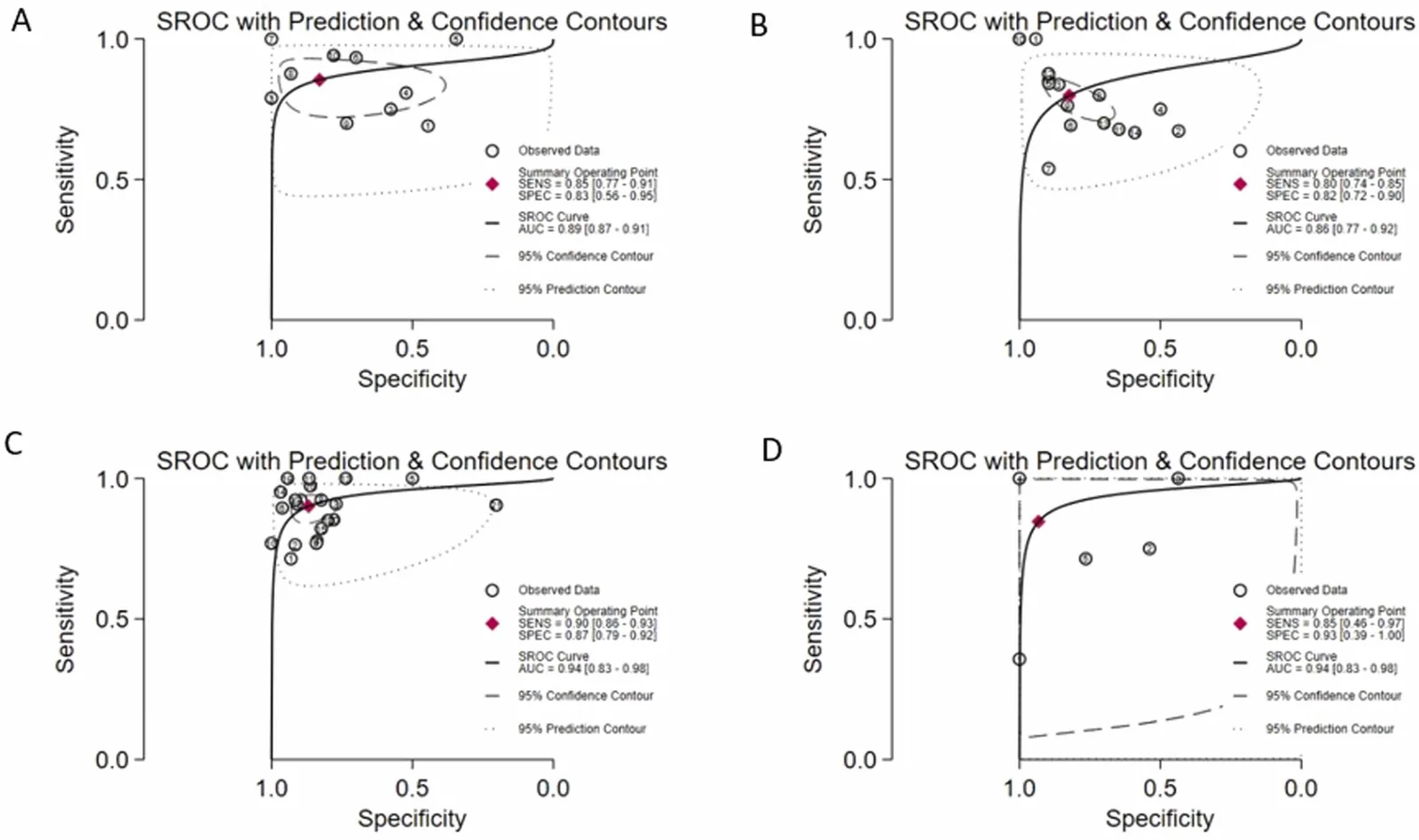
Summary receiver operating characteristic curves of the main pulmonary artery Doppler parameters for predicting neonatal respiratory distress syndrome. Panel A shows the pulsatility index (PI), Panel B shows the resistance index (RI), Panel C shows the acceleration time-to-ejection time ratio (AT/ET), and Panel D shows peak systolic velocity (PSV). Circles represent study-specific estimates, diamonds represent the summary operating points, dashed contours indicate the 95% confidence regions, dotted contours indicate the 95% prediction regions, and the solid line represents the summary receiver operating characteristic curve.

A total of 10 studies involving 1833 pregnant women, using cutoff values ranging from 1.79 to 2.47, were included in this pooled analysis [17,21,23,[27], [28], [29],^32^,^35^,^37^,^39^]. Pooled analysis showed a sensitivity of 85% (95% CI: 77–91%), a specificity of 83% (95% CI: 56–95%), a LR+ of 5.0 (95% CI: 1.6–15.6), a LR- of 0.18 (95% CI: 0.10–0.30), a DOR of 28.6 (95% CI: 6.1–132.8), and an AUC of 0.89 (95% CI: 0.87–0.91). A high level of heterogeneity was observed (I^2^ = 95%) (Fig. 2A).

### 3.4 Diagnostic performance of fetal pulmonary artery Doppler for RI

A total of 15 studies involving 2302 pregnant women, using cutoff values ranging from 0.740 to 0.900, were included in this pooled analysis [8,17,18,21,23,[27], [28], [29], [30],^32^,^34^,^35^,[37], [38], [39]]. Pooled analysis showed a sensitivity of 80% (95% CI: 74–85%), a specificity of 82% (95% CI: 72–90%), a LR + of 4.5 (95% CI: 2.6–8.0), a LR- of 0.24 (95% CI: 0.17–0.35), a DOR of 19 (95% CI: 7–46), and an AUC of 0.86 (95% CI: 0.77–0.92). A high level of heterogeneity was observed (I^2^ = 98%) (Fig. 2B).

### 3.5 Diagnostic performance of fetal pulmonary artery Doppler for AT/ET

A total of 21 studies involving 2882 pregnant women, using cutoff values ranging from 0.210 to 0.478, were included in this pooled analysis [16,17,[19], [20], [21],^23^,[25], [26], [27], [28], [29],[32], [33], [34], [35], [36], [37], [38], [39], [40], [41]]. Pooled analysis showed a sensitivity of 90% (95% CI: 86–93%), a specificity of 87% (95% CI: 79–92%), a LR+ of 6.9 (95% CI: 4.3–10.9), a LR- of 0.11 (95% CI: 0.08–0.16), a DOR of 60 (95% CI: 32.1–112.3), and an AUC of 0.94 (95% CI: 0.83–0.98). A high level of heterogeneity was observed (I^2^ = 97%) (Fig. 2**C**).

### 3.6 Diagnostic performance of fetal pulmonary artery Doppler for S/D

Only two studies evaluating fetal pulmonary artery Doppler S/D were identified, involving 200 pregnant women and using cutoff values ranging from 6.93 to 7.00 [28,32]. Sensitivity ranged from 53.85% to 59.60%, while specificity ranged from 54.40% to 60.92%. A pooled analysis was not performed because only two studies were available.

### 3.7 Diagnostic performance of fetal pulmonary artery Doppler for PSV

A total of 5 studies involving 425 pregnant women, using cutoff values ranging from 70.0 to 78.5 cm/s, were included in this pooled analysis [16,21,28,32,34]. Pooled analysis showed a sensitivity of 85% (95% CI: 46–97%), a specificity of 93% (95% CI: 39–100%), a LR+ of 12.2 (95% CI: 0.8–193.1), a LR- of 0.17 (95% CI: 0.04–0.77), a DOR of 73.4 (95% CI: 2.8–1921.1), and an AUC of 0.94 (95% CI: 0.83–0.98). A high level of heterogeneity was observed (I^2^ = 94%) (Fig. 2D).

### 3.8 Diagnostic performance of fetal pulmonary artery Doppler for MV

Two studies evaluating fetal pulmonary artery Doppler MV were identified, involving 105 pregnant women [16,34]. Only one study [34] reported the cutoff values of 25.5 cm/s for predicting NRDS. Sensitivity ranged from 21.4% to 70.0%, while specificity ranged from 65.0% to 96.6%. A pooled analysis was not performed because only two studies were available.

### 3.9 Fagan’s nomogram

**Fig. 3:**
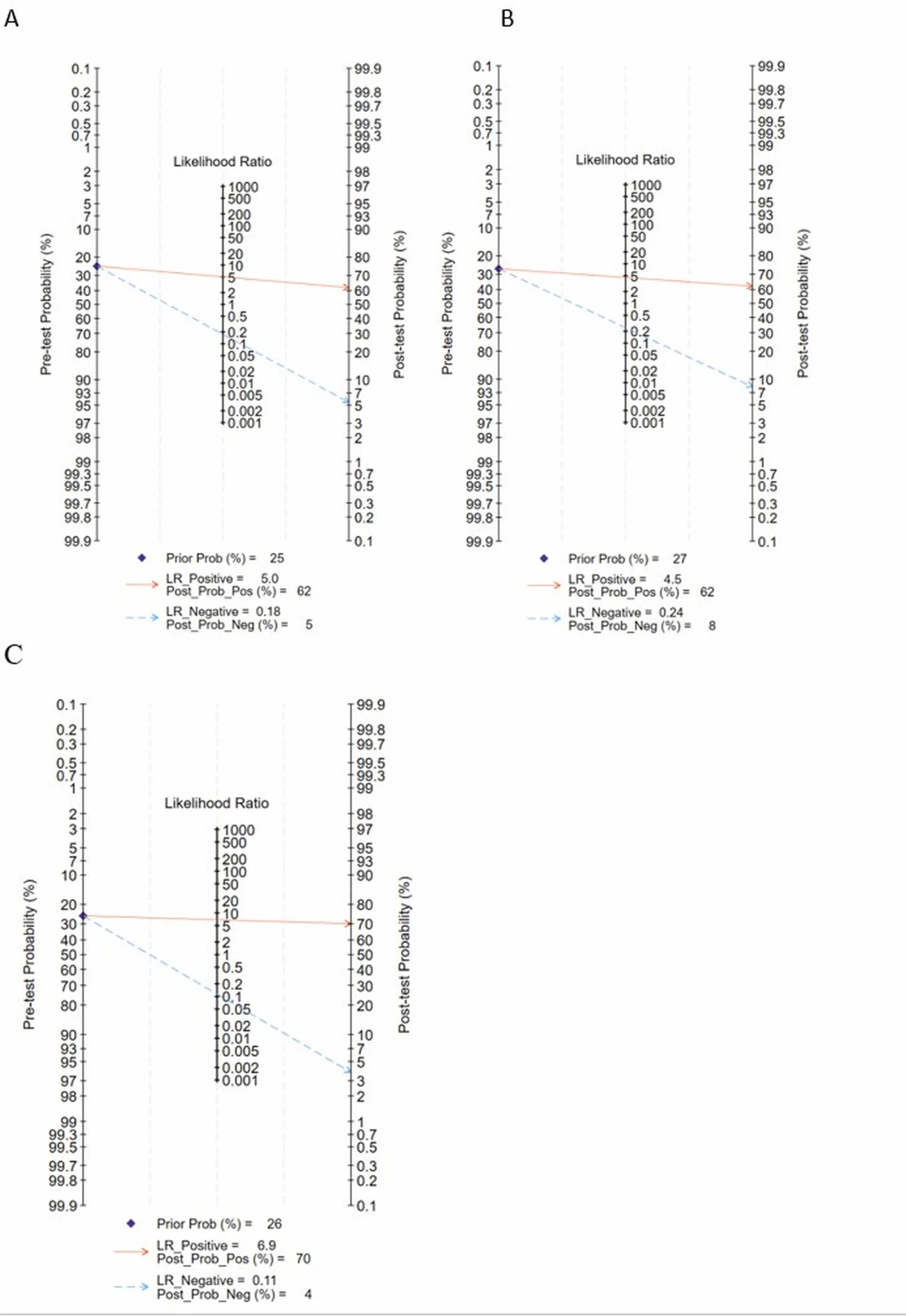
Fagan nomograms showing changes from pre-test to post-test probabilities for predicting neonatal respiratory distress syndrome using the main pulmonary artery Doppler parameters. Panel A shows the pulsatility index (PI), Panel B shows the resistance index (RI), and Panel C shows the acceleration time-to-ejection time ratio (AT/ET). The pre-test probability for each parameter was derived from the verified pooled event rate among studies contributing to the corresponding marker-specific diagnostic meta-analysis. Final pooled likelihood ratios from the bivariate random-effects models were used to estimate the corresponding post-test probabilities.

Fagan's nomogram was applied to three fetal pulmonary artery Doppler parameters to estimate post-test probabilities of NRDS. For PI, assuming a pretest probability of 25%, an LR+ of 5.0 increased the post-test probability to approximately 62%, whereas an LR− of 0.18 reduced it to approximately 5%. For RI, assuming a pretest probability of 27%, an LR+ of 4.5 increased the post-test probability to approximately 62%, whereas an LR− of 0.24 reduced it to approximately 8%. For AT/ET, assuming a pretest probability of 26%, an LR+ of 6.9 increased the post-test probability to approximately 70%, whereas an LR− of 0.11 reduced it to approximately 4%. Among the three parameters, AT/ET demonstrated the highest post-test probability following a positive test result and the lowest post-test probability following a negative test result (Fig. 3).

### 3.10 Continuous outcome of fetal pulmonary artery parameters

Across continuous outcome analyses, PI and RI values were significantly higher in fetuses whose neonates developed NRDS than in those without NRDS. Because mean differences (MD) were calculated as non-RDS minus RDS, these findings corresponded to pooled MDs of −0.15 (95% CI: −0.25 to −0.04; p = 0.01; I² = 95.04%) across 20 studies [[15], [16], [17],[19], [20], [21],[23], [24], [25], [26], [27], [28], [29],^32^,[34], [35], [36], [37],^39^,^41^] (Supplementary Figure S1), and −0.04 (95% CI: −0.07 to −0.01; p = 0.003; I² = 98.73%) across 26 studies [8,[15], [16], [17], [18], [19], [20], [21], [22], [23], [24], [25], [26], [27], [28], [29], [30], [31], [32],[34], [35], [36], [37], [38], [39],^41^] (Supplementary Figure S2), respectively.

In contrast, AT/ET, PSV, and MV were significantly lower in fetuses whose neonates developed NRDS. The corresponding pooled MDs were 0.06 (95% CI 0.05–0.08; p < 0.001; I² = 96.37%) for AT/ET across 23 studies [[15], [16], [17],[19], [20], [21],[23], [24], [25], [26], [27], [28], [29],[32], [33], [34], [35], [36], [37], [38], [39], [40], [41]] (Supplementary Figure S3), 4.02 (95% CI 1.93–6.11; p < 0.001; I² = 84.68%) for PSV across 13 studies [[15], [16], [17],^19^,^21^,^25^,[27], [28], [29],^32^,^34^,^35^,^39^] (Supplementary Figure S4), and 4.63 (95% CI 3.03–6.24; p < 0.001; I² = 0%) for MV across 3 studies [16,19,34] (Supplementary Figure S5). No statistically significant between-group differences were observed for S/D (MD −0.02, 95% CI −0.19–0.16; p = 0.86; I² = 51.03% across 7 studies [15,17,25,28,29,32,39]; Supplementary Figure S6) or EDV (MD 0.83, 95% CI −0.23–1.89; p = 0.12; I² = 52.42% across 4 studies [16,19,29,34]; Supplementary Figure S7). No studies reported continuous outcome data for PAP.

### 3.11 Subgroup, sensitivity analyses, and meta-regression

Subgroup analyses were performed for parameters with ten or more studies (PI, RI, and AT/ET for diagnostic outcomes; PI, RI, AT/ET, and PSV for continuous outcomes), stratified by fetal population category (preterm, mixed, or term), geographic region (Egypt versus Non-Egypt). For diagnostic outcomes, subgroup analysis by population type showed that AT/ET maintained consistently high diagnostic performance across preterm, mixed, and term populations, with a modest reduction in heterogeneity (I² = 74.30%, 64.80%, and 73.60%, respectively) (Supplementary Figure S8.1-S8.3). Subgroup analyses for PI and RI in the available fetal population strata showed persistent heterogeneity (Supplementary Figure S9-S10.2). For continuous outcomes, subgroup analyses restricted to preterm populations showed directionally consistent differences for PI, RI, AT/ET, and PSV (Supplementary Figure S11–14).

Subgroup analysis by geographic region (Egypt only) showed directionally consistent estimates for PI (I² = 91.10%) and AT/ET (I² = 67.80%) (Supplementary Figures S15–S16). Exclusion of the single case series [33] did not materially alter the pooled AT/ET estimate (I² = 68.30%).

Sensitivity analyses remained directionally consistent after exclusion of influential studies, although the magnitude of pooled estimates changed for some parameters. AT/ET estimates remained comparatively stable (Supplementary Figures S17–23).

Meta-regression incorporating fetal population category, geographic region, sample size, NRDS prevalence, and antenatal corticosteroid use identified no statistically significant moderator of heterogeneity for any Doppler parameter. However, these analyses were exploratory and may have had limited statistical power because of the small number of studies contributing to each parameter-specific model. Therefore, the absence of statistically significant moderators should not be interpreted as evidence that true effect modifiers were absent.

### 3.12 Risk of bias

**Fig. 4:**
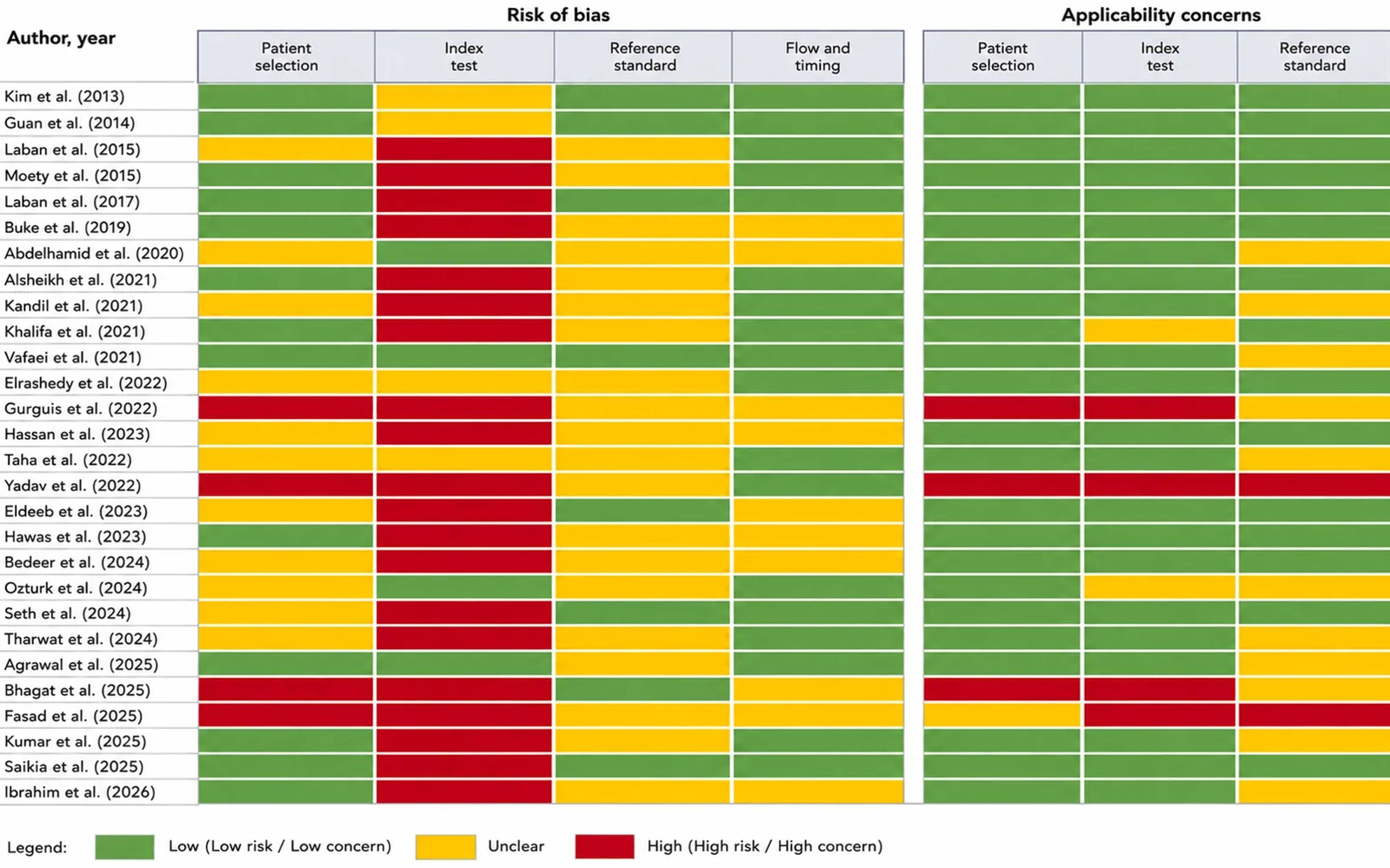
QUADAS-2 traffic-light plot of risk of bias and applicability concerns across the included studies.

The QUADAS-2 assessment showed variable risk of bias profiles across domains (Fig. 4). For patient selection, 13 studies were rated as low risk, 11 as unclear risk, and 4 as high risk. The index test domain represented the principal methodological concern, with 20 studies rated as high risk, 4 as unclear risk, and 4 as low risk. For the reference standard domain, 20 studies were rated as unclear risk and 8 as low risk. Flow and timing were rated as low risk in 18 studies and unclear risk in 10 studies. Applicability concerns were generally low, with low concern ratings in 24 studies for patient selection, 22 studies for the index test, and 15 studies for the reference standard, although several studies had unclear or high applicability concerns.

### 3.13 Publication bias

Publication bias was assessed using Deeks' funnel plot asymmetry test for PI, RI, AT/ET and PSV. No statistically significant asymmetry was detected for any parameter (Supplementary Figures S24.1–S24.4). However, the PSV analysis should be interpreted cautiously because only five studies contributed to this parameter.

## 4 Discussion

### 4.1 Main findings

This SRMA evaluated the diagnostic performance of fetal pulmonary artery Doppler parameters for predicting NRDS across 28 studies involving 3576 pregnant women. Among the evaluated parameters, AT/ET demonstrated the strongest and most consistent evidence, with an AUC of 0.94 and relatively stable diagnostic performance across gestational age strata. PSV also yielded a numerically high AUC of 0.94; however, this estimate was based on only five studies and was accompanied by substantial uncertainty. The wide confidence intervals for the likelihood ratio and diagnostic odds ratio, including an LR + confidence interval that crossed 1, substantially limit confidence in the magnitude and reproducibility of its diagnostic performance. Therefore, the numerical similarity in AUC between PSV and AT/ET should not be interpreted as indicating comparable evidentiary strength. Among the other parameters with adequate evidence for pooled analysis, PI achieved an AUC of 0.89 and RI an AUC of 0.86, whereas evidence for S/D, EDV, and MV remained insufficient for pooled diagnostic accuracy analysis.

These findings align with fetal pulmonary vascular physiology: pulmonary vascular resistance normally falls as lung expansion increases pulmonary blood flow after birth [42]. Fetuses who developed NRDS showed higher PI and RI, consistent with elevated resistance and impaired vascular adaptation, and lower AT/ET, reflecting delayed pulmonary arterial flow acceleration and incomplete lung maturation [10].

Between-study heterogeneity was substantial across the diagnostic and continuous outcome analyses, with I² frequently exceeding 90%, and likely reflects differences in gestational age, study populations, NRDS definitions, Doppler acquisition methods, operator experience, and ultrasound equipment. Sensitivity analyses generally preserved the direction of the principal findings, although some pooled estimates changed after exclusion of influential studies. Considerable variation in study-specific cutoff values, potentially reflecting differences in gestational age, baseline NRDS risk, acquisition technique, equipment, and threshold-derivation methods, likely contributed further by altering the sensitivity-specificity balance. Given this variation and the substantial residual heterogeneity, a universal cutoff cannot currently be recommended; gestational age-specific or population-specific thresholds require prospective derivation and external validation using standardized protocols.

### 4.2 Comparison with existing literature

Compared with other predictors of NRDS, the lecithin-to-sphingomyelin ratio and lamellar body count have shown relatively high diagnostic performance in previous studies. For lamellar body count, reported sensitivity ranged from 59% to 99% and specificity from 65% to 97%, whereas the lecithin-to-sphingomyelin ratio showed sensitivity ranging from 73% to 96% and specificity from 80% to 92% [7]. However, the clinical use of these biomarkers remains limited because of their invasiveness. Among non-invasive alternatives, fetal lung volume has shown sensitivity of 46.0–97.3% and specificity of 65.91–95.70% [8,30,35,43], though its applicability is limited by wide cutoff variation and few studies. Quantitative fetal lung texture analysis evaluates image-based tissue characteristics and has also shown promising performance, with a multicenter study reporting a sensitivity of 74.3% and specificity of 88.6% [9,44,45]. In contrast, fetal pulmonary artery Doppler provides physiological information related to pulmonary vascular resistance and maturation. These approaches may therefore offer complementary structural, texture-based, and vascular information rather than represent directly competing diagnostic modalities. However, direct comparisons of diagnostic performance should be interpreted cautiously because the available evidence is derived from studies with differing populations, gestational age distributions, NRDS definitions, diagnostic thresholds, and methodological designs.

### 4.3 Clinical implications

Despite favorable pooled diagnostic performance, substantial residual heterogeneity currently limits routine clinical implementation of fetal pulmonary artery Doppler for NRDS prediction. AT/ET appears the most promising parameter, but prospective multicenter validation using standardized acquisition protocols is required before clinical use.

### 4.4 Research implications

Future studies should adopt standardized Doppler acquisition protocols and prospectively record antenatal corticosteroid exposure and the interval from corticosteroid administration to Doppler assessment and delivery. Gestational age-specific or population-specific cutoff values should be prospectively derived and externally validated in independent populations. Further studies should evaluate whether combining pulmonary artery Doppler with complementary structural or texture-based sonographic biomarkers, such as fetal lung volumetry or quantitative lung texture analysis, improves diagnostic performance beyond individual imaging parameters. Future research should also include clinically relevant high-risk populations, such as pregnancies complicated by gestational diabetes mellitus or fetal growth restriction.

### 4.5 Strengths and limitations

Principal strengths include adherence to PRISMA-DTA guidelines, use of the bivariate random-effects model with HSROC analysis, comprehensive evaluation of diagnostic and continuous outcomes across 28 studies and 3576 participants, systematic heterogeneity exploration, and application of Fagan's nomogram for direct clinical translation of likelihood ratio estimates.

Several limitations warrant acknowledgment. Substantial residual heterogeneity persisted across major analyses, precluding a universal cutoff recommendation. Meta-regression identified no statistically significant moderator, but the limited number of studies per parameter-specific model reduced statistical power, so clinically important effect modifiers, including GA, population characteristics, corticosteroid exposure, acquisition protocols, equipment, operator technique, and NRDS definitions, may have remained undetected. Most included studies originated from Egypt and India with varying acquisition protocols, which may limit generalizability and technical reproducibility, and interobserver and intraobserver reproducibility was reported in only two studies, limiting assessment of measurement reliability.

## 5 Conclusions

Fetal pulmonary artery Doppler, particularly AT/ET, PI, and RI, shows promising diagnostic performance for NRDS, most consistently for AT/ET across gestational age. PSV showed a high but imprecise AUC; evidence for S/D, EDV, and MV remained insufficient for pooling. Substantial heterogeneity and the lack of a validated cutoff limit implementation, pending standardized multicenter validation and clarifying corticosteroid effects.

## CRediT authorship contribution statement

**Potsanop Kassayanan:** Writing – review & editing, Writing – original draft, Validation, Methodology, Investigation, Formal analysis, Data curation. **Kasidis Nontaprom:** Writing – original draft, Visualization, Software, Investigation, Data curation. **Atiwit Threeratananont:** Methodology, Investigation. **Nathaporn Thitinan:** Investigation. **Monchai Suntipap:** Writing – review & editing, Writing – original draft, Visualization, Validation, Supervision, Methodology, Formal analysis, Conceptualization.

## Declaration of Generative AI and AI-assisted technologies in the writing process

During the preparation of this work, the authors used Claude (Anthropic, San Francisco, CA, USA) to assist with language editing. After using this tool, the authors reviewed and edited all content as needed and take full responsibility for the content of the published article.

## Funding statement

This research did not receive any specific grant from funding agencies in the public, commercial, or not-for-profit sectors.

## Declaration of Competing Interest

The authors declare that they have no competing interests.
