# Supplementary material for: Role of fetal pulmonary artery Doppler parameters for predicting neonatal respiratory distress syndrome: A systematic review and meta-analysis of diagnostic accuracy
Source: Eur J Obstet Gynecol Reprod Biol X. 2026 Sep 16;32:100494. doi: 10.1016/j.eurox.2026.100494 (PMC13595148; doi:10.1016/j.eurox.2026.100494)
Supplement: Supplementary file 1 [file mmc1.docx]

**Supplementary Table S1.1.** Medline search strategy

| Domain | Subdomain | Query | Search term | Number |
| --- | --- | --- | --- | --- |
| Population (P) | P1 pregnant women | #1 | Pregnancy | 1,217,324 |
|  |  | #2 | Pregnant women | 176,673 |
|  |  | #3 | Pregnant people | 30,164 |
|  |  | #4 | #1 OR #2 OR #3 | 1,235,516 |
|  | P2 Neonates | #5 | Preterm | 115,176 |
|  |  | #6 | Preterm birth | 96,861 |
|  |  | #7 | Premature birth | 75,360 |
|  |  | #8 | Preterm fetus | 11,582 |
|  |  | #9 | Preterm labor | 46,784 |
|  |  | #10 | Low gestational age | 38,453 |
|  |  | #11 | Term | 1,705,443 |
|  |  | #12 | Term birth | 63,868 |
|  |  | #13 | Term fetus | 28,466 |
|  |  | #14 | Term labor | 174,404 |
|  |  | #15 | Newborn | 904,319 |
|  |  | #16 | Neonate | 918,191 |
|  |  | #17 | #5 OR #6 OR #7 OR #8 OR #9 OR #10 OR #11 OR #12 OR #13 OR #14 OR #15 OR #16 | 2,669,483 |
| **Total P** | P1+P2 | #18 | #4 AND #17 | 387,838 |
|  |  |  |  |  |
| Index test (I) | I1 Pulmonary artery doppler index | #19 | “Pulmonary artery” | 90,166 |
|  |  | #20 | Doppler | 164,783 |
|  |  | #21 | Flow | 1,074,393 |
|  |  | #22 | pulsatility index | 10,419 |
|  |  | #23 | resistance index | 103,202 |
|  |  | #24 | acceleration time-to-ejection time ratio | 40 |
|  |  | #25 | systolic-to-diastolic ratio | 273 |
|  |  | #26 | peak systolic velocity | 12,100 |
|  |  | #27 | end diastolic velocity | 7,521 |
|  |  | #28 | mean velocity | 53,432 |
|  |  | #29 | pulmonary artery pressure | 37,715 |
|  |  | #30 | #19 AND (#20 OR #21 OR #22 OR #23 OR #24 OR #25 OR #26 OR #27 OR #28 OR #29) | 40,843 |
|  |  |  |  |  |
|  | I2 ultrasonography | #31 | Ultrasound | 2,249,778 |
|  |  | #32 | Ultrasonography | 2,053,228 |
|  |  |  |  |  |
|  |  | #33 | #31 OR #32 | 2,249,816 |
| Total I | I1+I2 | #34 | #30 AND #33 | 11,460 |
| Reference test (R) |  |  |  |  |
| Diagnosis of interest (D) | D1 Respiratory distress syndrome | #35 | “Respiratory distress syndrome” | 62,643 |
| Total |  | #36 | #18 AND #33 AND #35 | 646 |

**Supplementary Table S1.2**. Scopus search strategy

| Domain | Subdomain | Query | Search term | Number |
| --- | --- | --- | --- | --- |
| Population (P) | P1 pregnant women | #1 | ALL(Pregnancy) | 2,151,913 |
|  |  | #2 | ALL(Pregnant women) | 629,435 |
|  |  | #3 | ALL(Pregnant people) | 121,118 |
|  |  | #4 | #1 OR #2 OR #3 | 2,324,384 |
|  | P2 Neonates | #5 | ALL(Preterm) | 443,476 |
|  |  | #6 | ALL(Preterm birth) | 304,528 |
|  |  | #7 | ALL(Premature birth) | 244,918 |
|  |  | #8 | ALL(Preterm fetus) | 104,018 |
|  |  | #9 | ALL(Preterm labor) | 98,232 |
|  |  | #10 | ALL(Low gestational age) | 218,091 |
|  |  | #11 | ALL(Term) | 15,604,645 |
|  |  | #12 | ALL(Term birth) | 686,499 |
|  |  | #13 | ALL(Term fetus) | 220,619 |
|  |  | #14 | ALL(Term labor) | 496,782 |
|  |  | #15 | ALL(Newborn) | 1,578,399 |
|  |  | #16 | ALL(Neonate) | 543,691 |
|  |  | #17 | #5 OR #6 OR #7 OR #8 OR #9 OR #10 OR #11 OR #12 OR #13 OR #14 OR #15 OR #16 | 16,961,907 |
| **Total P** | P1+P2 | #18 | #4 AND #17 | 1,096,153 |
|  |  |  |  |  |
| Index test (I) | I1 Pulmonary artery doppler index | #19 | ALL(“Pulmonary artery”) | 240,274 |
|  |  | #20 | ALL(Doppler) | 840,793 |
|  |  | #21 | ALL(Flow) | 10,219,173 |
|  |  | #22 | ALL(pulsatility index) | 31,802 |
|  |  | #23 | ALL(resistance index) | 2,075,849 |
|  |  | #24 | ALL(acceleration time-to-ejection time ratio) | 186 |
|  |  | #25 | ALL(systolic-to-diastolic ratio) | 736 |
|  |  | #26 | ALL(peak systolic velocity) | 29,957 |
|  |  | #27 | ALL(end diastolic velocity) | 28,581 |
|  |  | #28 | ALL(mean velocity) | 586,057 |
|  |  | #29 | ALL(pulmonary artery pressure) | 202,602 |
|  |  | #30 | #19 AND (#20 OR #21 OR #22 OR #23 OR #24 OR #25 OR #26 OR #27 OR #28 OR #29) | 144,750 |
|  |  |  |  |  |
|  | I2 ultrasonography | #31 | ALL(Ultrasound) | 1,955,314 |
|  |  | #32 | ALL(Ultrasonography) | 620,941 |
|  |  |  |  |  |
|  |  | #33 | #31 OR #32 | 2,164,965 |
| Total I | I1+I2 | #34 | #30 AND #33 | 23,603 |
| Reference test (R) |  |  |  |  |
| Diagnosis of interest (D) | O1 Respiratory distress syndrome | #35 | ALL(“Respiratory distress syndrome”) | 247,194 |
| Total |  | #36 | #18 AND #33 AND #35 | 414 |

**Supplementary Table S1.3:** Embase search strategy

| Domain | Subdomain | Query | Search term | Number |
| --- | --- | --- | --- | --- |
| Population (P) | P1 pregnant women | #1 | 'pregnancy'/exp | 997,990 |
|  |  | #2 | pregnan*:ti,ab,kw | 964,607 |
|  |  | #3 | 'pregnant women':ti,ab,kw | 196,924 |
|  |  | #4 | 'pregnant people':ti,ab,kw | 2,137 |
|  |  | #5 | #1 OR #2 OR #3 OR #4 | 1,350,104 |
|  | P2 Neonates | #6 | 'premature birth'/exp | 172,710 |
|  |  | #7 | 'newborn'/exp | 751,462 |
|  |  | #8 | 'neonate'/exp | 751,462 |
|  |  | #9 | preterm:ti,ab,kw | 167,836 |
|  |  | #10 | 'preterm birth':ti,ab,kw | 46,719 |
|  |  | #11 | 'premature birth':ti,ab,kw | 9,299 |
|  |  | #12 | 'preterm fetus':ti,ab,kw | 231 |
|  |  | #13 | 'preterm labor':ti,ab,kw | 12,316 |
|  |  | #14 | 'low gestational age':ti,ab,kw | 1,548 |
|  |  | #15 | term:ti,ab,kw | 2,353,001 |
|  |  | #16 | 'term birth':ti,ab,kw | 3,805 |
|  |  | #17 | 'term fetus':ti,ab,kw | 474 |
|  |  | #18 | 'term labor':ti,ab,kw | 1,402 |
|  |  | #19 | newborn:ti,ab,kw | 214,238 |
|  |  | #20 | neonate*:ti,ab,kw | 177,439 |
|  |  | #21 | #6 OR #7 OR #8 OR #9 OR #10 OR #11 OR #12 OR #13 OR #14 OR #15 OR #16 OR #17 OR #18 OR #19 OR #20 | 3,230,897 |
| **Total P** | P1+P2 | #22 | #5 AND #21 | 343,589 |
| Index test (I) | I1 Pulmonary artery doppler index | #23 | 'pulmonary artery'/exp | 71,705 |
|  |  | #24 | 'pulmonary artery':ti,ab,kw | 100,538 |
|  |  | #25 | doppler:ti,ab,kw | 196,339 |
|  |  | #26 | flow:ti,ab,kw | 1,353,557 |
|  |  | #27 | 'pulsatility index':ti,ab,kw | 10,506 |
|  |  | #28 | 'resistance index':ti,ab,kw | 15,129 |
|  |  | #29 | 'acceleration time to ejection time ratio':ti,ab,kw | 30 |
|  |  | #30 | 'systolic-to-diastolic ratio':ti,ab,kw | 104 |
|  |  | #31 | 'peak systolic velocity':ti,ab,kw | 8,078 |
|  | I2 ultrasonography | #32 | 'end diastolic velocity':ti,ab,kw | 2,996 |
|  |  | #33 | 'mean velocity':ti,ab,kw | 5,549 |
|  |  | #34 | 'pulmonary artery pressure':ti,ab,kw | 21,968 |
|  |  | #35 | (#23 OR #24) AND (#25 OR #26 OR #27 OR #28 OR #29 OR #30 OR #31 OR #32 OR #33 OR #34) | 39,061 |
|  |  | #36 | 'ultrasonography'/exp | 1,262,471 |
|  |  | #37 | ultrasound:ti,ab,kw | 627,307 |
|  |  | #38 | ultrasonography:ti,ab,kw | 189,915 |
|  |  | #39 | #36 OR #37 OR #38 | 1,608,456 |
| Total I | I1+I2 | #40 | #35 AND #39 | 15,136 |
| Reference test (R) |  | #41 | 'respiratory distress syndrome'/exp | 125,260 |
|  |  | #42 | 'respiratory distress syndrome':ti,ab,kw | 63,650 |
| Diagnosis of interest (D) | O1 Respiratory distress syndrome | #43 | #41 OR #42 | 137,161 |
| Total |  | #44 | #22 AND #40 AND #43 | 35 |

**Supplementary Table S2.** Excluded full text articles and reasons for exclusion

| **No.** | **Study** | **Article title** | **Primary reason for exclusion** | **Additional details** |
| --- | --- | --- | --- | --- |
| 1 | Gast et al. (1995) | [Ultrasound diagnosis of fetal lung maturity] | Not reported outcome of interests | Not reported NRDS outcome |
| 2 | Azpurua et al. (2010) | Acceleration/ejection time ratio in the fetal pulmonary artery predicts fetal lung maturity | Not reported outcome of interests | Not reported NRDS outcome |
| 3 | Schenone et al. (2014) | Predicting fetal lung maturity using the fetal pulmonary artery Doppler wave acceleration/ejection time ratio | Not reported outcome of interests | Not reported NRDS outcome |
| 4 | Wang et al. (2016) | Prenatal assessment of pulmonary maturity on 3-D ultrasound | Not reported outcome of interests | Not reported NRDS outcome |
| 5 | Gungor et al. (2017) | Effect of Betamethasone on Fetal Pulmonary and Umbilical Artery Doppler Velocimetry and Relationship  With Respiratory Distress Syndrome Development | Not exposure of interests | Not main pulmonary artery |
| 6 | Sule et al. (2021) | Effects of SARS‐COV‐2 infection on fetal pulmonary artery Doppler parameters | Not reported outcome of interests | Not reported NRDS outcome |
| 7 | Han et al. (2022) | Clinical Analysis of Fetal Lung Development Index and Pregnancy Outcome in Pregnant Women with Gestational Diabetes Mellitus with Satisfactory Blood Glucose Control | Not reported outcome of interests | Not reported NRDS outcome |
| 8 | Hosseinzadeh et al. (2022) | Relationship Between Peak Systolic Velocity in Pulmonary Artery Color Doppler and Neonatal Respiratory Outcomes in Fetal Growth Restriction With Abnormal Fetoplacental Circulation: A Prospective Cohort Study | Not reported outcome of interests | Not reported NRDS outcome |
| 9 | Li et al. (2022) | The Role and Clinical Value of Optimized Fetal Main Pulmonary Artery Doppler Parameters in the Diagnosis and Prognosis Monitoring of Neonatal Respiratory Distress Syndrome | Retracted article |  |
| 10 | Wang et al. (2022) | Acceleration time to Ejection time ratio in fetal pulmonary artery system can predict neonatal respiratory disorders in gestational diabetic mellitus women | Not reported outcome of interests | Composite outcome |
| 11 | Beser et al. (2023) | Assessment of fetal pulmonary artery Doppler indices in pregnant women with rheumatoid arthritis and ankylosing spondylitis | Not reported outcome of interests | Not reported NRDS outcome |
| 12 | Oluklu et al. (2023) | Effect of maternal autoimmune diseases on fetal pulmonary artery Doppler indices: A case-control study | Not reported outcome of interests | Not reported NRDS outcome, only report correlation. |
| 13 | Abbas et al. (2024) | Fetal pulmonary artery Doppler's predictive usefulness in newborn respiratory distress syndrome | Potential duplicate publication | This study appeared to contain duplicated data with Bedeer et al. (2024). However, after consideration of the methodology, Bedeer et al. (2024) was selected as the primary report for data extraction. |
| 14 | Amjadi et al. (2024) | Association of maternal serum vitamin D level with fetal pulmonary artery Doppler indices and neonatal respiratory distress syndrome | Not reported outcome of interests | Insufficient continuous data for pooling outcomes (not report standard deviation) |
| 15 | Bulan et al. (2025) | Pulmonary Vascular Doppler and Fetal Lung Biometry as Predictors of Neonatal Respiratory Complications in Early- and Late-Onset Fetal Growth Restriction | Not reported outcome of interests | Composite outcome |
| 16 | Wang et al. (2025) | Assessment of fetal lung maturity and prediction of neonatal respiratory distress syndrome by MV-Flow imaging | Not exposure of interests | Study of microvascular flow imaging |
| 17 | Yildirim et al. (2025) | Fetal Pulmonary Artery Doppler Evaluation in Pregnant Women with Behçet’s Disease: A Case-Control Study | Not reported outcome of interests | Not reported NRDS outcome |

Abbreviations: NRDS, neonatal respiratory distress syndrome.

**Supplementary Table S3.** Study-level diagnostic accuracy data

| Doppler marker | Author, year | | Number of participants (NRDS/non-NRDS) | Cutoff | TP | FP | FN | TN | Reported sensitivity  (%) | Reported specificity (%) |
| --- | --- | --- | --- | --- | --- | --- | --- | --- | --- | --- |
| **Pulsatility index** | | | | | | | | | | |
|  | | Moety et al. (2015) [17] | 55 / 643 | 2.150 | 38 | 357 | 17 | 286 | 69.10 | 44.50 |
|  | | Alsheikh et al. (2021) [21] | 16 / 104 | 1.790 | 12 | 44 | 4 | 60 | 75.00 | 57.70 |
|  | | Khalifa et al. (2021) [23] | 38 / 105 | 2.330 | 30 | 0 | 8 | 105 | 79.00 | 100.00 |
|  | | Hassan et al. (2023) [27] | 26 / 44 | 2.470 | 21 | 21 | 5 | 23 | 80.80 | 52.30 |
|  | | Taha et al. (2022) [28] | 13 / 87 | 2.000 | 13 | 57 | 0 | 30 | 100.00 | 34.48 |
|  | | Yadav et al. (2022) [29] | 15 / 60 | 2.180 | 14 | 18 | 1 | 42 | 93.30 | 70.00 |
|  | | Bedeer et al. (2024) [32] | 11 / 89 | 2.350 | 11 | 0 | 0 | 89 | 100.00 | 100.00 |
|  | | Tharwat et al. (2024) [35] | 113 / 87 | 2.195 | 99 | 6 | 14 | 81 | 87.60 | 93.10 |
|  | | Bhagat et al. (2025) [37] | 20 / 30 | 1.900 | 14 | 8 | 6 | 22 | 70.00 | 72.00 |
|  | | Kumar et al. (2025) [39] | 68 / 209 | 2.160 | 64 | 46 | 4 | 163 | 93.78 | 77.94 |
| **Resistance index** | | | | | | | | | | |
|  | | Laban et al. (2015) [8] | 11 / 69 | 0.740 | 11 | 4 | 0 | 65 | 100.00 | 94.20 |
|  | | Moety et al. (2015) [17] | 55 / 643 | 0.770 | 37 | 363 | 18 | 280 | 67.30 | 43.50 |
|  | | Laban et al. (2017) [18] | 37 / 43 | 0.770 | 31 | 6 | 6 | 37 | 83.33 | 85.26 |
|  | | Alsheikh et al. (2021) [21] | 16 / 104 | 0.850 | 12 | 52 | 4 | 52 | 75.00 | 50.00 |
|  | | Khalifa et al. (2021) [23] | 38 / 105 | 0.890 | 32 | 11 | 6 | 94 | 84.20 | 89.50 |
|  | | Hassan et al. (2023) [27] | 26 / 44 | 0.8962 | 18 | 8 | 8 | 36 | 69.20 | 81.80 |
|  | | Taha et al. (2022) [28] | 13 / 87 | 0.870 | 7 | 9 | 6 | 78 | 53.85 | 89.66 |
|  | | Yadav et al. (2022) [29] | 15 / 60 | 0.860 | 12 | 17 | 3 | 43 | 80.00 | 71.67 |
|  | | Eldeeb et al. (2023) [30] | 59 / 88 | 0.750 | 45 | 15 | 14 | 73 | 76.27 | 82.95 |
|  | | Bedeer et al. (2024) [32] | 11 / 89 | 0.785 | 11 | 0 | 0 | 89 | 100.00 | 100.00 |
|  | | Seth et al. (2024) [34] | 28 / 34 | 0.900 | 19 | 12 | 9 | 22 | 67.00 | 65.00 |
|  | | Tharwat et al. (2024) [35] | 113 / 87 | 0.855 | 99 | 9 | 14 | 78 | 87.60 | 89.70 |
|  | | Bhagat et al. (2025) [37] | 20 / 30 | 0.810 | 14 | 9 | 6 | 21 | 68.00 | 70.00 |
|  | | Fasad et al. (2025) [38] | 12 / 88 | 0.765 | 8 | 36 | 4 | 52 | 68.70 | 59.00 |
|  | | Kumar et al. (2025) [39] | 68 / 209 | 0.800 | 58 | 22 | 10 | 187 | 85.17 | 89.71 |
| **Acceleration time-to-ejection time ratio** | | | | | | | | | | |
|  | | Guan et al. (2014) [16] | 14 / 29 | NR | 10 | 2 | 4 | 27 | 71.40 | 93.10 |
|  | | Moety et al. (2015) [17] | 55 / 643 | 0.305 | 42 | 54 | 13 | 589 | 76.40 | 91.60 |
|  | | Buke et al. (2019) [19] | 22 / 83 | 0.327 | 20 | 19 | 2 | 64 | 90.90 | 77.10 |
|  | | Abdelhamid et al. (2020) [20] | 9 / 31 | 0.300 | 7 | 5 | 2 | 26 | 77.78 | 82.87 |
|  | | Alsheikh et al. (2021) [21] | 16 / 104 | 0.320 | 16 | 52 | 0 | 52 | 100.00 | 50.00 |
|  | | Khalifa et al. (2021) [23] | 38 / 105 | 0.300 | 34 | 4 | 4 | 101 | 89.50 | 96.20 |
|  | | Elrashedy et al. (2022) [25] | 13 / 87 | 0.310 | 12 | 9 | 1 | 78 | 95.00 | 90.00 |
|  | | Guirguis et al. (2022) [26] | 26 / 34 | 0.300 | 24 | 6 | 2 | 28 | 92.30 | 82.40 |
|  | | Hassan et al. (2023) [27] | 26 / 44 | 0.305 | 20 | 7 | 6 | 37 | 76.90 | 84.10 |
|  | | Taha et al. (2022) [28] | 13 / 87 | 0.250 | 10 | 0 | 3 | 87 | 76.92 | 100.00 |
|  | | Yadav et al. (2022) [29] | 15 / 60 | 0.300 | 15 | 8 | 0 | 52 | 98.33 | 86.67 |
|  | | Bedeer et al. (2024) [32] | 11 / 89 | 0.346 | 10 | 8 | 1 | 81 | 94.40 | 90.90 |
|  | | Ozturk et al. (2024) [33] | 6 / 19 | 0.478 | 6 | 5 | 0 | 14 | 94.00 | 72.20 |
|  | | Seth et al. (2024) [34] | 28 / 34 | 0.290 | 23 | 6 | 5 | 28 | 82.00 | 82.00 |
|  | | Tharwat et al. (2024) [35] | 113 / 87 | 0.298 | 110 | 12 | 3 | 75 | 97.30 | 86.20 |
|  | | Agrawal et al. (2025) [36] | 39 / 131 | 0.300 | 36 | 11 | 3 | 120 | 92.30 | 91.60 |
|  | | Bhagat et al. (2025) [37] | 20 / 30 | 0.210 | 17 | 6 | 3 | 24 | 85.00 | 80.00 |
|  | | Fasad et al. (2025) [38] | 12 / 88 | 0.295 | 12 | 5 | 0 | 83 | 100.00 | 94.20 |
|  | | Kumar et al. (2025) [39] | 68 / 209 | 0.240 | 58 | 46 | 10 | 163 | 85.65 | 77.94 |
|  | | Saikia et al. (2025) [40] | 82 / 93 | 0.287 | 78 | 3 | 4 | 90 | 95.70 | 96.30 |
|  | | Ibrahim et al. (2026) [41] | 21 / 148 | 0.267 | 19 | 118 | 2 | 30 | 90.50 | 20.30 |
| **Systolic-to-diastolic ratio** | | | | | | | | | | |
|  | | Taha et al. (2022) [28] | 13 / 87 | 7.00 | 7 | 34 | 6 | 53 | 53.85 | 60.92 |
|  | | Bedeer et al. (2024) [32] | 11 / 89 | 6.93 | 7 | 41 | 4 | 48 | 59.60 | 54.40 |
| **Peak systolic velocity** | | | | | | | | | | |
|  | | Guan et al. (2014) [16] | 14 / 29 | NR | 5 | 0 | 9 | 29 | 35.70 | 100.00 |
|  | | Alsheikh et al. (2021) [21] | 16 / 104 | 78.50 | 12 | 48 | 4 | 56 | 75.00 | 53.80 |
|  | | Taha et al. (2022) [28] | 13 / 87 | 70.00 | 13 | 49 | 0 | 38 | 100.00 | 43.68 |
|  | | Bedeer et al. (2024) [32] | 11/89 | 74.45 | 11 | 0 | 0 | 89 | 100.00 | 100.00 |
|  | | Seth et al. (2024) [34] | 28/34 | 73.00 | 20 | 8 | 8 | 26 | 70.00 | 75.00 |
| **Mean velocity** | | | | | | | | | | |
|  | | Guan et al. (2014) [16] | 14 / 29 | NR | 3 | 1 | 11 | 28 | 21.40 | 96.60 |
|  | | Seth et al. (2024) [34] | 28 / 34 | 25.50 | 20 | 12 | 8 | 22 | 70.00 | 65.00 |

Abbreviations: AT/ET, acceleration time to ejection time ratio; EDV, end-diastolic velocity; MV, mean velocity; NR, not reported; NRDS, neonatal respiratory distress syndrome; PI, pulsatility index; PSV, peak systolic velocity; RI, resistive index; S/D, systolic-to-diastolic ratio; TP, true positive; FP, false positive; FN, false negative; TN, true negative; ND, not derivable. Sensitivity and specificity are values reported in the original studies. When complete 2 × 2 contingency data were not directly reported, TP, FP, FN, and TN were reconstructed by back-calculation from the reported diagnostic accuracy measures and available group sizes. Because integer cell counts were required for meta-analysis, reconstructed 2 × 2 data may not exactly reproduce the reported sensitivity and specificity values.

**Supplementary Table S4.** Study-level continuous Doppler values

| Doppler marker | Author, year | Number of participants | Number of non-NRDS | Mean ± S.D. of non-NRDS | Number of NRDS | Mean ± S.D. of NRDS |
| --- | --- | --- | --- | --- | --- | --- |
| **Pulsatility index** |  |  |  |  |  |  |
|  | Kim et al. (2013) [15] | 42 | 31 | 2.08 ± 0.20 | 11 | 2.07 ± 0.17 |
|  | Guan et al. (2014) [16] | 43 | 29 | 2.60 ± 0.19 | 14 | 2.64 ± 0.26 |
|  | Moety et al. (2015) [17] | 698 | 643 | 2.18 ± 0.23 | 55 | 2.27 ± 0.23 |
|  | Buke et al. (2019) [19] | 105 | 83 | 1.94 ± 0.90 | 22 | 1.73 ± 0.45 |
|  | Abdelhamid et al. (2020) [20] | 40 | 31 | 2.43 ± 0.48 | 9 | 2.24 ± 0.56 |
|  | Alsheikh et al. (2021) [21] | 120 | 104 | 1.74 ± 0.23 | 16 | 1.90 ± 0.20 |
|  | Khalifa et al. (2021) [23] | 143 | 105 | 1.96 ± 0.20 | 38 | 2.51 ± 0.33 |
|  | Vafaei et al. (2021) [24] | 25 | 17 | 2.09 ± 0.18 | 8 | 2.03 ± 0.20 |
|  | Elrashedy et al. (2022) [25] | 100 | 87 | 1.81 ± 0.38 | 13 | 2.09 ± 0.30 |
|  | Guirguis et al. (2022) [26] | 60 | 34 | 2.45 ± 0.54 | 26 | 2.27 ± 0.74 |
|  | Hassan et al. (2023) [27] | 70 | 44 | 2.43 ± 0.03 | 26 | 2.67 ± 0.19 |
|  | Taha et al. (2022) [28] | 100 | 87 | 2.18 ± 0.19 | 13 | 2.04 ± 0.22 |
|  | Yadav et al. (2022) [29] | 75 | 60 | 2.02 ± 0.27 | 15 | 2.29 ± 0.21 |
|  | Bedeer et al. (2024) [32] | 100 | 89 | 2.07 ± 0.14 | 11 | 2.52 ± 0.05 |
|  | Seth et al. (2024) [34] | 62 | 34 | 2.50 ± 0.20 | 28 | 2.50 ± 0.19 |
|  | Tharwat et al. (2024) [35] | 200 | 87 | 1.90 ± 0.30 | 113 | 2.60 ± 0.30 |
|  | Agrawal et al. (2025) [36] | 170 | 131 | 1.99 ± 0.39 | 39 | 1.87 ± 0.34 |
|  | Bhagat et al. (2025) [37] | 50 | 30 | 1.78 ± 0.18 | 20 | 2.01 ± 0.22 |
|  | Kumar et al. (2025) [39] | 277 | 209 | 1.89 ± 0.23 | 68 | 2.25 ± 0.21 |
|  | Ibrahim et al. (2026) [41] | 169 | 148 | 1.57 ± 0.24 | 21 | 1.62 ± 0.26 |
| **Resistance index** |  |  |  |  |  |  |
|  | Kim et al. (2013) [15] | 42 | 31 | 0.90 ± 0.02 | 11 | 0.89 ± 0.03 |
|  | Guan et al. (2014) [16] | 43 | 29 | 0.92 ± 0.02 | 14 | 0.92 ± 0.03 |
|  | Laban et al. (2015) [8] | 80 | 69 | 0.67 ± 0.01 | 11 | 0.92 ± 0.04 |
|  | Moety et al. (2015) [17] | 698 | 643 | 0.76 ± 0.09 | 55 | 0.80 ± 0.11 |
|  | Laban et al. (2017) [18] | 80 | 43 | 0.73 ± 0.07 | 37 | 0.84 ± 0.06 |
|  | Buke et al. (2019) [19] | 105 | 83 | 0.81 ± 0.27 | 22 | 0.73 ± 1.41 |
|  | Abdelhamid et al. (2020) [20] | 40 | 31 | 0.82 ± 0.11 | 9 | 0.77 ± 0.13 |
|  | Alsheikh et al. (2021) [21] | 120 | 104 | 0.83 ± 0.06 | 16 | 0.88 ± 0.05 |
|  | Kandil et al. (2021) [22] | 120 | 101 | 0.74 ± 0.02 | 19 | 0.80 ± 0.05 |
|  | Khalifa et al. (2021) [23] | 143 | 105 | 0.84 ± 0.01 | 38 | 0.90 ± 0.03 |
|  | Vafaei et al. (2021) [24] | 25 | 17 | 0.83 ± 0.05 | 8 | 0.79 ± 0.04 |
|  | Elrashedy et al. (2022) [25] | 100 | 87 | 0.83 ± 0.07 | 13 | 0.88 ± 0.05 |
|  | Guirguis et al. (2022) [26] | 60 | 34 | 0.81 ± 0.13 | 26 | 0.78 ± 0.13 |
|  | Hassan et al. (2023) [27] | 70 | 44 | 0.88 ± 0.01 | 26 | 0.90 ± 0.01 |
|  | Taha et al. (2022) [28] | 100 | 87 | 0.76 ± 0.08 | 13 | 0.74 ± 0.12 |
|  | Yadav et al. (2022) [29] | 75 | 60 | 0.79 ± 0.08 | 15 | 0.87 ± 0.07 |
|  | Eldeeb et al. (2023) [30] | 147 | 88 | 0.73 ± 0.04 | 59 | 0.81 ± 0.06 |
|  | Hawas et al. (2023) [31] | 200 | 174 | 0.83 ± 0.05 | 26 | 0.82 ± 0.08 |
|  | Bedeer et al. (2024) [32] | 100 | 89 | 0.75 ± 0.01 | 11 | 0.82 ± 0.01 |
|  | Seth et al. (2024) [34] | 62 | 34 | 0.91 ± 0.02 | 28 | 0.89 ± 0.02 |
|  | Tharwat et al. (2024) [35] | 200 | 87 | 0.80 ± 0.20 | 113 | 0.90 ± 0.05 |
|  | Agrawal et al. (2025) [36] | 170 | 131 | 0.83 ± 0.09 | 39 | 0.83 ± 0.08 |
|  | Bhagat et al. (2025) [37] | 50 | 30 | 0.79 ± 0.04 | 20 | 0.83 ± 0.05 |
|  | Fasad et al. (2025) [38] | 100 | 88 | 0.75 ± 0.03 | 12 | 0.79 ± 0.04 |
|  | Kumar et al. (2025) [39] | 277 | 209 | 0.75 ± 0.07 | 68 | 0.86 ± 0.05 |
|  | Ibrahim et al. (2026) [41] | 169 | 148 | 0.81 ± 0.04 | 21 | 0.81 ± 0.04 |
| **Acceleration time-to-ejection time ratio** |  |  |  |  |  |  |
|  | Kim et al. (2013) [15] | 42 | 31 | 0.31 ± 0.06 | 11 | 0.35 ± 0.05 |
|  | Guan et al. (2014) [16] | 43 | 29 | 0.24 ± 0.01 | 14 | 0.17 ± 0.02 |
|  | Moety et al. (2015) [17] | 698 | 643 | 0.33 ± 0.07 | 55 | 0.21 ± 0.05 |
|  | Buke et al. (2019) [19] | 105 | 83 | 0.39 ± 0.07 | 22 | 0.30 ± 0.04 |
|  | Abdelhamid et al. (2020) [20] | 40 | 31 | 0.34 ± 0.05 | 9 | 0.27 ± 0.05 |
|  | Alsheikh et al. (2021) [21] | 120 | 104 | 0.31 ± 0.07 | 16 | 0.26 ± 0.03 |
|  | Khalifa et al. (2021) [23] | 143 | 105 | 0.35 ± 0.04 | 38 | 0.24 ± 0.04 |
|  | Vafaei et al. (2021) [24] | 25 | 17 | 0.26 ± 0.03 | 8 | 0.24 ± 0.29 |
|  | Elrashedy et al. (2022) [25] | 100 | 87 | 0.41 ± 0.11 | 13 | 0.26 ± 0.03 |
|  | Guirguis et al. (2022) [26] | 60 | 34 | 0.30 ± 0.03 | 26 | 0.23 ± 0.04 |
|  | Hassan et al. (2023) [27] | 70 | 44 | 0.35 ± 0.04 | 26 | 0.30 ± 0.03 |
|  | Taha et al. (2022) [28] | 100 | 87 | 0.35 ± 0.04 | 13 | 0.28 ± 0.05 |
|  | Yadav et al. (2022) [29] | 75 | 60 | 0.34 ± 0.03 | 15 | 0.24 ± 0.06 |
|  | Bedeer et al. (2024) [32] | 100 | 89 | 0.39 ± 0.02 | 11 | 0.33 ± 0.01 |
|  | Ozturk et al. (2024) [33] | 25 | 19 | 0.28 ± 0.11 | 6 | 0.36 ± 0.17 |
|  | Seth et al. (2024) [34] | 62 | 34 | 0.31 ± 0.03 | 28 | 0.26 ± 0.03 |
|  | Tharwat et al. (2024) [35] | 200 | 87 | 0.30 ± 0.10 | 113 | 0.20 ± 0.10 |
|  | Agrawal et al. (2025) [36] | 170 | 131 | 0.33 ± 0.04 | 39 | 0.27 ± 0.04 |
|  | Bhagat et al. (2025) [37] | 50 | 30 | 0.23 ± 0.02 | 20 | 0.20 ± 0.02 |
|  | Fasad et al. (2025) [38] | 100 | 88 | 0.32 ± 0.02 | 12 | 0.27 ± 0.01 |
|  | Kumar et al. (2025) [39] | 277 | 209 | 0.28 ± 0.04 | 68 | 0.21 ± 0.04 |
|  | Saikia et al. (2025) [40] | 175 | 93 | 0.34 ± 0.06 | 82 | 0.24 ± 0.03 |
|  | Ibrahim et al. (2026) [41] | 169 | 148 | 0.25 ± 0.01 | 21 | 0.25 ± 0.01 |
| **Systolic-to-diastolic ratio** |  |  |  |  |  |  |
|  | Kim et al. (2013) [15] | 42 | 31 | 11.35 ± 2.42 | 11 | 10.26 ± 2.32 |
|  | Moety et al. (2015) [17] | 698 | 643 | 6.86 ± 0.29 | 55 | 6.92 ± 0.29 |
|  | Elrashedy et al. (2022) [25] | 100 | 87 | 10.26 ± 3.60 | 13 | 13.33 ± 5.57 |
|  | Taha et al. (2022) [28] | 100 | 87 | 6.90 ± 0.40 | 13 | 6.96 ± 0.42 |
|  | Yadav et al. (2022) [29] | 75 | 60 | 8.09 ± 1.55 | 15 | 8.19 ± 0.17 |
|  | Bedeer et al. (2024) [32] | 100 | 89 | 7.11 ± 0.37 | 11 | 6.94 ± 0.29 |
|  | Kumar et al. (2025) [39] | 277 | 209 | 4.78 ± 1.25 | 68 | 4.85 ± 1.41 |
| **Peak systolic velocity** |  |  |  |  |  |  |
|  | Kim et al. (2013) [15] | 42 | 31 | 112.15 ± 37.20 | 11 | 119.30 ± 53.79 |
|  | Guan et al. (2014) [16] | 43 | 29 | 85.68 ± 9.01 | 14 | 69.91 ± 14.87 |
|  | Moety et al. (2015) [17] | 698 | 643 | 67.21 ± 4.80 | 55 | 65.05 ± 5.33 |
|  | Buke et al. (2019) [19] | 105 | 83 | 60.28 ± 22.96 | 22 | 57.90 ± 21.19 |
|  | Alsheikh et al. (2021) [21] | 120 | 104 | 77.40 ± 14.30 | 16 | 88.10 ± 12.00 |
|  | Elrashedy et al. (2022) [25] | 100 | 87 | 79.21 ± 18.55 | 13 | 92.43 ± 12.15 |
|  | Hassan et al. (2023) [27] | 70 | 44 | 66.27 ± 4.43 | 26 | 62.03 ± 4.55 |
|  | Taha et al. (2022) [28] | 100 | 87 | 67.95 ± 5.88 | 13 | 55.46 ± 5.85 |
|  | Yadav et al. (2022) [29] | 75 | 60 | 79.95 ± 12.46 | 15 | 77.69 ± 0.62 |
|  | Bedeer et al. (2024) [32] | 100 | 89 | 76.64 ± 0.51 | 11 | 73.02 ± 0.35 |
|  | Seth et al. (2024) [34] | 62 | 34 | 77.20 ± 8.40 | 28 | 67.00 ± 10.10 |
|  | Tharwat et al. (2024) [35] | 200 | 87 | 57.80 ± 19.20 | 113 | 52.40 ± 14.80 |
|  | Kumar et al. (2025) [39] | 277 | 209 | 60.69 ± 10.99 | 68 | 59.82 ± 13.02 |
| **End diastolic velocity** |  |  |  |  |  |  |
|  | Guan et al. (2014) [16] | 43 | 29 | 7.36 ± 2.25 | 14 | 6.19 ± 2.22 |
|  | Buke et al. (2019) [19] | 105 | 83 | 15.87 ± 7.02 | 22 | 13.71 ± 5.60 |
|  | Yadav et al. (2022) [29] | 75 | 60 | 11.63 ± 3.53 | 15 | 9.88 ± 2.92 |
|  | Seth et al. (2024) [34] | 62 | 34 | 6.81 ± 1.05 | 28 | 6.82 ± 0.96 |
| **Mean velocity** |  |  |  |  |  |  |
|  | Guan et al. (2014) [16] | 43 | 29 | 30.22 ± 3.64 | 14 | 24.66 ± 5.45 |
|  | Buke et al. (2019) [19] | 105 | 83 | 36.10 ± 23.10 | 22 | 35.27 ± 20.74 |
|  | Seth et al. (2024) [34] | 62 | 34 | 28.32 ± 4.35 | 28 | 24.05 ± 3.58 |

Abbreviations: AT/ET, acceleration time to ejection time ratio; EDV, end-diastolic velocity; MV, mean velocity; NRDS, neonatal respiratory distress syndrome; PI, pulsatility index; PSV, peak systolic velocity; RI, resistive index; S/D, systolic-to-diastolic ratio; S.D., standard deviation.

**Supplementary Figure S1.** Continuous outcome of pulsatility index

**Supplementary Figure S2.** Continuous outcome of resistance index

**Supplementary Figure S3.** Continuous outcome of acceleration time-to-ejection time ratio

**Supplementary Figure S4.** Continuous outcome of peak systolic velocity

**Supplementary Figure S5.** Continuous outcome of mean velocity

**Supplementary Figure S6.** Continuous outcome of systolic-to-diastolic ratio

**Supplementary Figure S7.** Continuous outcome of end diastolic velocity

**Supplementary Figure S8.1.** Subgroup analyses of acceleration time-to-ejection time ratio by fetal population (preterm group)

**Supplementary Figure S8.2.** Subgroup analyses of acceleration time-to-ejection time ratio by fetal population (mixed preterm and term group)

**Supplementary Figure S8.3.** Subgroup analyses of acceleration time-to-ejection time ratio by fetal population (term group)

**Supplementary Figure S9.** Subgroup analyses of pulsatility index by fetal population (mixed preterm and term group)

**Supplementary Figure S10.1.** Subgroup analyses of resistance index by fetal population (mixed preterm and term group)

**Supplementary Figure S10.2.** Subgroup analyses of resistance index by fetal population (term group)

**Supplementary Figure S11.** Continuous outcome subgroup analysis of pulsatility index by fetal population *(*preterm group***)***

**Supplementary Figure S12.** Continuous outcome subgroup analysis of resistance index by fetal population *(*preterm group***)***

**Supplementary Figure S13.** Continuous outcome subgroup analysis of the acceleration time-to-ejection time ratio by fetal population *(*preterm group***)***

**Supplementary Figure S14.** Continuous outcome subgroup analysis of the peak systolic velocity by fetal population *(*preterm group***)***

**Supplementary Figure S15.** Subgroup analyses of pulsatility index by geographic region (only Egypt)

**Supplementary Figure S16.** Subgroup analyses of acceleration time-to-ejection time ratio by geographic region (only Egypt)

**Supplementary Figure S17.** Sensitivity analysis after exclusion of outlier for pulsatility index

**Supplementary Figure S18.** Sensitivity analysis after exclusion of outlier for resistance index

**Supplementary Figure S19.** Sensitivity analysis after exclusion of outlier for acceleration time-to-ejection time ratio

**Supplementary Figure S20.** Sensitivity analysis after exclusion of outlier for continuous outcome of pulsatility index

**Supplementary Figure S21.** Sensitivity analysis after exclusion of outlier for continuous outcome of resistance index

**Supplementary Figure S22.** Sensitivity analysis after exclusion of outlier for continuous outcome of acceleration time-to-ejection time ratio

**Supplementary Figure S23.** Sensitivity analysis after exclusion of outlier for continuous outcome of peak systolic velocity

**Supplementary Figure S24.1.** Deeks’ funnel plots for assessment of publication bias of pulsatility index

**Supplementary Figure S24.2.** Deeks’ funnel plots for assessment of publication bias of resistance index

**Supplementary Figure S24.3.** Deeks’ funnel plots for assessment of publication bias of the acceleration time-to-ejection time ratio

**Supplementary Figure S24.4.** Deeks’ funnel plots for assessment of publication bias of peak systolic velocity
